# Calcium Dynamics in Astrocyte-Neuron Communication from Intracellular to Extracellular Signaling

**DOI:** 10.3390/cells14211709

**Published:** 2025-10-31

**Authors:** Agnieszka Nowacka, Maciej Śniegocki, Ewa A. Ziółkowska

**Affiliations:** 1 Department of Neurosurgery, Nicolas Copernicus University in Toruń, Collegium Medicum in Bydgoszcz, ul. Curie Skłodowskiej 9, 85-094 Bydgoszcz, Poland; sniegocki@cm.umk.pl; 2 Department of Pediatrics, School of Medicine, Washington University in St. Louis, St. Louis, MO 63110, USA

**Keywords:** astrocytes, calcium signaling, neuron-glia communication, Intracellular Ca^2+^ dynamics, extracellular calcium ([Ca^2+^]o)

## Abstract

Astrocytic calcium signaling is a central mechanism of neuron-glia communication that operates across multiple spatial and temporal scales. Traditionally, research has focused on intracellular Ca^2+^ oscillations that regulate gliotransmitter release, ion homeostasis, and metabolic support. Recent evidence, however, reveals that extracellular calcium ([Ca^2+^]o) is not a passive reservoir but a dynamic signaling mediator capable of influencing neuronal excitability within milliseconds. Through mechanisms such as calcium-sensing receptor (CaSR) activation, ion channel modulation, surface charge effects, and ephaptic coupling, astrocytes emerge as active partners in both slow and rapid modes of communication. This dual perspective reshapes our understanding of brain physiology and disease. Disrupted Ca^2+^ signaling contributes to network instability in epilepsy, synaptic dysfunction in Alzheimer’s and Parkinson’s disease, and impaired maturation in neurodevelopmental disorders. Methodological advances, including Ca^2+^-selective microelectrodes, genetically encoded extracellular indicators, and computational modeling, are beginning to uncover the richness of extracellular Ca^2+^ dynamics, though challenges remain in achieving sufficient spatial and temporal resolution. By integrating classical intracellular pathways with emerging insights into extracellular signaling, this review highlights astrocytes as central architects of the ionic landscape. Recognizing calcium as both an intracellular messenger and an extracellular signaling mediator provides a unifying framework for neuron–glia interactions and opens new avenues for therapeutic intervention.

## 1. Introduction

Astrocytes, once regarded as passive “glue,” are now recognized as dynamic regulators of brain activity [1,2]. A pivotal discovery came in 1990, when Cornell-Bell and colleagues demonstrated that astrocytes respond to neurotransmitters with intracellular calcium (Ca^2+^) elevations, establishing them as excitable partners in neural networks [2,3]. Since then, it has become clear that astrocytic Ca^2+^ signaling shapes synaptic efficacy and circuit function across multiple timescales [4,5].

Calcium is central to this transformation. Unlike neurons that rely on action potentials, astrocytes use graded Ca^2+^ dynamics to integrate synaptic input and modulate neuronal activity. These signals are generated through diverse mechanisms, including endoplasmic reticulum release via inositol 1,4,5-trisphosphate receptors (IP_3_R), store-operated calcium entry (SOCE), metabotropic glutamate and purinergic receptors, voltage-gated channels, and Na^+^-Ca^2+^ exchanger reversal during neurotransmitter uptake [6,7,8]. Local Ca^2+^ microdomains influence individual synapses, while global waves propagate through astrocytic networks, enabling modulation of neuronal excitability with both spatial precision and temporal flexibility [9]. Functionally, these dynamics govern gliotransmitter release, extracellular ion homeostasis, and metabolic support [10,11,12].

Aberrant astrocytic Ca^2+^ signaling is implicated in epilepsy, Alzheimer’s disease, and Parkinson’s disease, where it destabilizes circuits through dysregulated gliotransmission and impaired ion regulation [6,12]. These observations underscore Ca^2+^ as both a physiological mediator and a therapeutic target.

Most reviews have emphasized intracellular Ca^2+^ oscillations and their role in gliotransmission. Here, we take a broader view by integrating these classical insights with emerging evidence that extracellular Ca^2+^ itself can act as a rapid signaling mediator. Recent advances, including Ca^2+^-selective microelectrodes, in vivo imaging with genetically encoded indicators, and optogenetic manipulations, reveal that fluctuations in extracellular calcium ([Ca^2+^]o) occur on millisecond timescales and directly influence neuronal excitability through mechanisms such as CaSR activation, ion channel modulation, and ephaptic coupling [9].

This review therefore synthesizes the established knowledge of intracellular Ca^2+^ regulation with novel concepts of extracellular dynamics. By examining how astrocytes sculpt both intracellular signals and the extracellular ionic landscape, we aim to highlight a unifying framework in which astrocytes emerge as active architects of neural communication and potential targets for therapeutic intervention.

## 2. Intracellular Calcium Signaling in Astrocytes: Established Mechanisms

### 2.1. Sources of Astrocytic Ca^2+^ Elevations

Astrocytes rely on multiple mechanisms to generate cytosolic Ca^2+^ elevations that encode information relevant to neuron-glia communication. A major source is the endoplasmic reticulum (ER), where Ca^2+^ is released through inositol 1,4,5-trisphosphate receptors (IP_3_Rs) activated by G-protein-coupled receptors. In astrocytes, the principal GPCRs linked to Ca^2+^ mobilization include metabotropic glutamate receptors (mGluR1/5), purinergic P2Y_1_ and P2Y_2_ receptors, muscarinic M1/M3 acetylcholine receptors, adrenergic α_1_-receptors, and protease-activated receptors (PAR-1), all of which trigger phospholipase C–dependent IP_3_ production and subsequent ER Ca^2+^ release [4,6,7,8]. This store release is replenished by store-operated calcium entry (SOCE), a process coordinated by STIM (stromal interaction molecule) sensors and Orai (Ca^2+^ release-activated Ca^2+^ channel protein) channels at ER-plasma membrane junctions [6,7]. In addition to these canonical pathways, astrocytes express metabotropic glutamate and purinergic P2Y receptors, both of which mobilize intracellular Ca^2+^ through phospholipase C–dependent IP_3_ production [8]. Ionotropic receptors are less abundant but can provide localized Ca^2+^ entry in fine processes, contributing to microdomain signaling [9]. Although voltage-gated calcium channels (VGCCs) are not as prominent as in neurons, evidence across brain regions indicates functional VGCC expression in subsets of astrocytes, where they contribute to depolarization-linked Ca^2+^ influx [6]. Another important route is the Na^+^-Ca^2+^ exchanger (NCX), which can operate in reverse when intracellular Na^+^ rises after neurotransmitter uptake, thereby driving additional Ca^2+^ entry independently of ER stores [13,14]. Together, these pathways provide astrocytes with a versatile toolkit for shaping intracellular calcium dynamics. The principal mechanisms, their modes of activation, and functional consequences are summarized in Table 1.

### 2.2. Spatial Organization of Ca^2+^ Signals

Astrocytic Ca^2+^ activity is highly compartmentalized, reflecting the complex morphology of these cells [15,16,17]. Local microdomains arise in thin perisynaptic processes where restricted geometry enables transient and spatially confined signals. Such microdomains can selectively influence nearby synapses without affecting distant compartments [18,19]. By contrast, global Ca^2+^ waves propagate through larger astrocytic territories, either via intracellular diffusion or through intercellular coupling [16,20]. Gap junctions composed of connexins allow Ca^2+^ waves to travel between astrocytes, while extracellular ATP released by one astrocyte can act on purinergic receptors of its neighbors to propagate signals across networks [9,10]. This dual organization enables astrocytes to operate at both local and global scales, fine-tuning synaptic transmission while also coordinating activity across broader brain regions.

### 2.3. Temporal Dynamics

Astrocytic Ca^2+^ signals are not uniform in time but span a wide spectrum of kinetics. Fast transients can occur within milliseconds to seconds and often follow bursts of neuronal activity, whereas slower components, including oscillations lasting minutes, shape long-term network states. Oscillatory patterns are particularly important because they can encode rhythmic information and influence neuronal excitability in a frequency-dependent manner [8]. Activity-dependent modulation ensures that astrocytic Ca^2+^ elevations scale with synaptic demand. For example, sensory stimulation in vivo evokes robust Ca^2+^ responses in cortical astrocytes, which are temporally aligned with neuronal firing and behavioral states [9]. The coexistence of fast, localized events with slower oscillations illustrates the flexibility of Ca^2+^ signaling in adapting to different modes of circuit operation.

### 2.4. Functional Consequences of Intracellular Ca^2+^

The functional consequences of astrocytic Ca^2+^ elevations are wide-ranging. One of the most studied outputs is gliotransmitter release. Elevations in intracellular Ca^2+^ can trigger exocytosis of glutamate, ATP, GABA, and D-serine, each exerting distinct effects on synaptic physiology [11,12]. For instance, Ca^2+^-dependent glutamate release enhances excitatory transmission via NMDA and metabotropic glutamate receptors, whereas ATP release can be converted to adenosine, which dampens synaptic activity and provides homeostatic control. These opposing influences highlight the bidirectional capacity of astrocytes to either amplify or suppress neuronal signaling depending on context. Beyond transmitter release, Ca^2+^ also regulates astrocytic metabolism, ion transport, and morphological plasticity [5,21,22]. By modulating Na^+^,K^+^-ATPase activity, astrocytes use Ca^2+^ as a signal to adjust extracellular potassium buffering, thereby stabilizing neuronal membrane potentials [10]. In parallel, Ca^2+^-dependent cytoskeletal remodeling contributes to dynamic changes in astrocytic processes that alter their engagement with synapses. Such multifunctional roles underscore why Ca^2+^ is considered a master regulator of astrocyte physiology and neuron-glia communication.

## 3. The Transition: From Intracellular to Extracellular Calcium Dynamics

### 3.1. Mechanisms Linking Intracellular Ca^2+^ to Extracellular Changes

Intracellular calcium dynamics in astrocytes are not confined to the cytosol, instead, they directly influence the composition of the extracellular milieu through a variety of transport and release mechanisms [5,17,23,24]. Among the most prominent are the Na^+^/Ca^2+^ exchangers (NCX and NCKX), which normally extrude Ca^2+^ in exchange for Na^+^ influx but can operate in reverse when intracellular Na^+^ rises during intense neurotransmitter uptake [25,26,27]. This reversal generates additional Ca^2+^ entry while simultaneously shaping extracellular calcium concentrations. Plasma membrane Ca^2+^-ATPases (PMCA) provide a complementary mechanism by actively exporting Ca^2+^ against its gradient, thereby contributing to the fine-tuning of perisynaptic calcium levels [28,29,30]. Voltage-gated calcium channels (VGCCs), although less abundant in astrocytes than in neurons, also participate in transmembrane fluxes. They allow for rapid influx during depolarization and, under certain conditions, mediate Ca^2+^ efflux when intracellular concentrations are elevated, further coupling astrocytic excitability to extracellular ion balance [16,31,32]. Beyond transporter, and channel-mediated fluxes, vesicular mechanisms provide an additional route linking intracellular and extracellular calcium. Vesicles enriched with Ca^2+^ can fuse with the plasma membrane during exocytosis, releasing their content directly into the extracellular space. Furthermore, astrocytes can secrete Ca^2+^-binding proteins through regulated exocytosis, effectively delivering molecules that alter local buffering capacity [6,13,33]. Together, these mechanisms ensure that intracellular Ca^2+^ elevations are rapidly translated into extracellular signals that neurons and neighboring glia can detect.

### 3.2. Extracellular Ca^2+^ Buffering Systems

The impact of astrocyte-driven calcium release depends not only on the flux itself but also on the buffering environment of the extracellular space. Calcium-binding proteins play a critical role in this regulation [4,16,22]. S100β, a member of the S100 family abundantly expressed in astrocytes, can be secreted into the extracellular milieu where it binds Ca^2+^ and participates in signaling through receptor for advanced glycation end-products (RAGE) [34,35,36]. Beyond buffering, extracellular S100β modulates neuronal growth, synaptic plasticity, and inflammatory responses, underscoring its dual role as a Ca^2+^-binding protein and signaling molecule. Other EF-hand (helix–loop–helix motif) Ca^2+^-binding proteins, such as calretinin and calbindin, traditionally studied in neurons, have also been detected in extracellular compartments where they contribute to shaping local calcium availability and signal propagation [37,38].

In parallel, the extracellular matrix (ECM) provides a structural scaffold with significant buffering capacity. Proteoglycans and glycosaminoglycans, with their high density of negative charges, can transiently bind divalent cations, effectively shaping diffusion profiles of Ca^2+^ in the extracellular space. Regional heterogeneity in matrix composition adds another layer of regulation: brain regions enriched in perineuronal nets may present different Ca^2+^ buffering properties compared to areas with more diffuse ECM organization [9,33,39,40]. These matrix-dependent differences help explain why extracellular calcium dynamics can vary across circuits despite similar astrocytic activity.

### 3.3. Spatial Constraints and Microenvironments

The translation of intracellular Ca^2+^ changes into extracellular signals is further sculpted by the geometry of neural microenvironments. In synaptic clefts, the restricted volume imposes strong constraints on diffusion, meaning that even modest Ca^2+^ fluxes from perisynaptic astrocytic processes can significantly alter local concentrations [16,22,41]. Such changes influence presynaptic release probability and postsynaptic excitability on a rapid timescale. The close apposition of fine astrocytic processes to excitatory synapses thus establishes a structural basis for astrocytes to act as modulators of extracellular calcium dynamics during ongoing synaptic activity [6,16,42]. Beyond synapses, soma–soma appositions offer another site of extracellular regulation. Recent work has described “satellite” astrocytes that envelop neuronal cell bodies, providing intimate contact zones where extracellular Ca^2+^ modulation can directly affect neuronal excitability [43,44]. The efficiency of this modulation depends not only on the molecular machinery involved but also on the geometry of the contact, with larger or more tightly sealed interfaces amplifying the impact of astrocytic Ca^2+^ handling.

Together, these findings highlight a transition in perspective: intracellular calcium signals in astrocytes are not isolated events but are continuously translated into extracellular changes through transporters, pumps, vesicular release, buffering proteins, and structural constraints. This interplay situates astrocytes as central architects of the extracellular calcium landscape, capable of influencing neuronal excitability and circuit function with both temporal precision and spatial specificity.

## 4. Extracellular Calcium as a Rapid Signaling Mediator

### 4.1. Evidence for Rapid [Ca^2+^]o Changes

For many years, astrocyte–neuron communication was framed almost exclusively in terms of gliotransmitter release. More recently, however, direct modulation of the extracellular calcium concentration ([Ca^2+^]o) has emerged as a complementary pathway, with evidence that [Ca^2+^]o can fluctuate on timescales previously considered unattainable for glia [14,42,45]. A critical advance has come from calcium-selective microelectrodes, which provide submillisecond resolution and reveal transient decreases in [Ca^2+^]o during bursts of synaptic activity. These drops occur locally in the synaptic cleft and recover quickly, indicating that astrocytic fluxes and neuronal uptake together can shape extracellular calcium on millisecond timescales [46,47,48]. Complementary in vivo imaging studies using genetically encoded extracellular calcium indicators have extended these observations to intact networks, showing that sensory stimulation and behavioral states are accompanied by rapid [Ca^2+^]o shifts in perisynaptic domains [49,50,51,52]. Optogenetic tools further strengthen causal links: when astrocytic Ca^2+^ pumps or exchangers are selectively activated or silenced, corresponding [Ca^2+^]o fluctuations can be observed in adjacent synapses [53,54,55,56]. Together, these methods converge on the conclusion that extracellular calcium is not a static reservoir but a dynamic signal capable of rapid modulation.

### 4.2. Mechanisms of Neuronal Sensitivity to [Ca^2+^]o

Neurons are exquisitely tuned to extracellular calcium, and small changes in [Ca^2+^]o can strongly influence excitability and synaptic function. One well-established pathway involves the calcium-sensing receptor (CaSR), a G-protein–coupled receptor expressed in subsets of neurons [44,57,58,59]. Activation of CaSR initiates intracellular cascades that regulate ion channel activity, neurotransmitter release, and gene expression, effectively coupling extracellular calcium availability to neuronal physiology [6,58,60,61].

Beyond CaSR, extracellular calcium acts directly on ion channels. Hyperpolarization-activated cyclic nucleotide-gated (HCN) channels are influenced by surface charge screening, where Ca^2+^ ions shield negative charges on the membrane, shifting voltage dependence and altering pacemaker currents [6,61,62]. Small- and large-conductance Ca^2+^-activated K^+^ channels (SK and BK) can also be modulated by extracellular calcium indirectly through its effects on local depolarization and intracellular Ca^2+^ entry. More speculative but increasingly supported are roles for the sodium leak channel NALCN, which has been proposed to respond to changes in [Ca^2+^]o through mechanisms involving auxiliary subunits [27,44,62,63,64].

Surface charge effects add another layer of regulation. Because Ca^2+^ is divalent, it can screen fixed negative charges on the outer leaflet of the neuronal membrane, shifting the gating properties of voltage-gated sodium and potassium channels [6,65]. Even modest [Ca^2+^]o reductions, on the order of hundreds of micromolar, can depolarize neuronal thresholds and increase excitability, illustrating how extracellular calcium dynamics translate into functional consequences for spike initiation and propagation [62,66].

**Figure 1:**
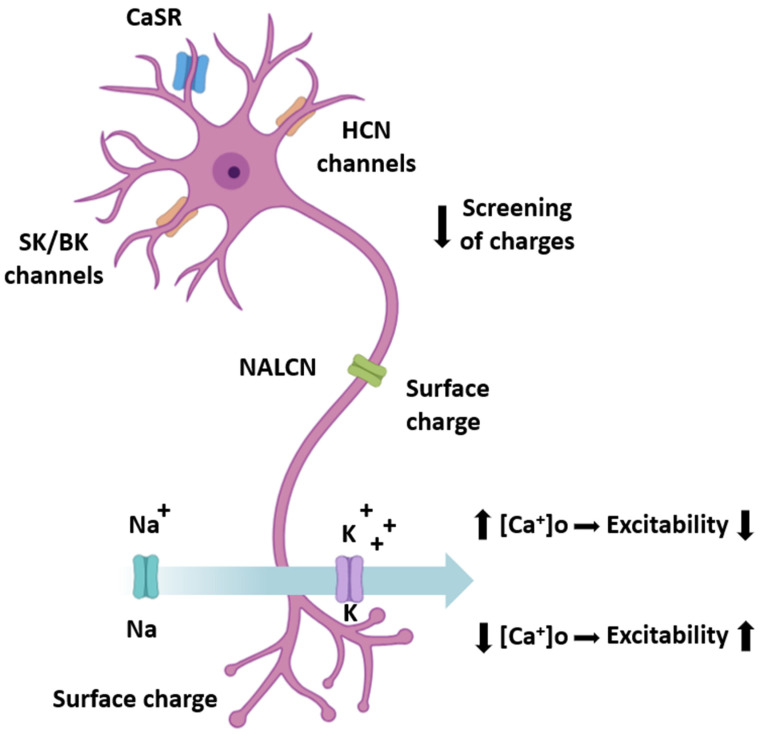
Neuronal mechanisms of sensitivity to extracellular calcium ([Ca^2+^]o).

The principal neuronal targets of extracellular calcium are summarized in Table 2, and their anatomical distribution and functional effects are schematically illustrated in Figure 1.

Schematic representation of a neuron illustrating major mechanisms of sensitivity to extracellular calcium ([Ca^2+^]o). Calcium-sensing receptors (CaSR) located on dendrites detect changes in [Ca^2+^]o and couple them to intracellular signaling cascades. Hyperpolarization-activated cyclic nucleotide-gated (HCN) channels and small- and large-conductance Ca^2+^-activated potassium channels (SK/BK) on dendrites are modulated by charge screening and local Ca^2+^ availability, influencing pacemaker activity and firing patterns. The sodium leak channel (NALCN) in the soma responds to changes in [Ca^2+^]o via auxiliary subunits, thereby affecting resting excitability. Surface charge effects of divalent Ca^2+^ on voltage-gated sodium and potassium channels alter their gating properties, shifting neuronal thresholds. Together, these mechanisms translate fluctuations in [Ca^2+^]o into changes in excitability, rhythmicity, and synaptic function. Abbreviations: CaSR—calcium-sensing receptor; HCN—hyperpolarization-activated cyclic nucleotide-gated channel; SK—small-conductance Ca^2+^-activated K^+^ channel; BK—big-conductance Ca^2+^-activated K^+^ channel; NALCN—sodium leak channel non-selective; NaV—voltage-gated sodium channel; KV—voltage-gated potassium channel.

### 4.3. Regional and Cell-Type Specificity

The sensitivity to [Ca^2+^]o is not uniform across the brain but exhibits pronounced cell-type and regional variation. Striatal cholinergic interneurons represent one of the most striking examples. Bennett and Wilson demonstrated that these cells show profound responsiveness to fluctuations in [Ca^2+^]o, a property amplified by their close association with satellite astrocytes that envelope the somata [40,67]. This arrangement creates restricted extracellular compartments where Ca^2+^ fluxes are particularly effective in shaping excitability.

Cortical pyramidal neurons also respond to local [Ca^2+^]o dynamics, with recent evidence suggesting that astrocytic processes in perisynaptic zones regulate activity-dependent calcium depletion during high-frequency firing [6,44,68]. In the brainstem, Morquette and colleagues have described neurons whose rhythmic activity is influenced by extracellular calcium changes, highlighting the diversity of mechanisms across regions. Comparative analysis suggests that [Ca^2+^]o-mediated modulation is a general phenomenon, but its exact role depends on both the structural configuration of astrocyte–neuron contacts and the intrinsic sensitivity of neuronal subtypes [6,10,42,69].

### 4.4. Functional Implications

The ability of extracellular calcium to act as a rapid signal carries important implications for network physiology. One domain is the generation and maintenance of network oscillations. Because [Ca^2+^]o shifts occur on millisecond scales, they can pace or reinforce rhythmic activity in the gamma and theta ranges. For example, periodic extracellular calcium dips can synchronize firing across populations by simultaneously modulating surface charge and ion channel gating. This ephaptic influence provides a non-synaptic mechanism for rhythm generation that complements synaptic inhibition and intrinsic pacemaker currents [6,33,66,70].

Synaptic plasticity represents another area where [Ca^2+^]o dynamics play a central role. On the presynaptic side, reduced [Ca^2+^]o can decrease release probability by limiting the driving force for Ca^2+^ entry, whereas elevated [Ca^2+^]o enhances release and supports short-term facilitation. Postsynaptically, fluctuations in extracellular calcium alter excitability by modulating NMDA receptor activity and voltage-gated channel thresholds. In this way, astrocyte-driven control of [Ca^2+^]o can directly affect the induction of long-term potentiation or depression [4,71,72,73]. Beyond presynaptic mechanisms, reduced extracellular calcium also exerts a direct impact on postsynaptic signaling. A lower [Ca^2+^]o diminishes the electrochemical driving force for Ca^2+^ entry through NMDA receptors and voltage-gated calcium channels, thereby attenuating the intracellular Ca^2+^ transients required for the induction of long-term potentiation (LTP) and long-term depression (LTD). Computational and theoretical studies by Nicolas Brunel, Michael Graupner, and others have shown that even modest decreases in extracellular calcium can shift the threshold for synaptic plasticity, altering the balance between potentiation and depression by limiting postsynaptic Ca^2+^ influx. These findings emphasize that extracellular calcium homeostasis, largely shaped by astrocytic activity, is a key determinant of synaptic learning rules and network adaptability [71,72].

Together, these findings reposition extracellular calcium from a background ion to an active signaling mediator. By combining rapid kinetics, multiple modes of neuronal sensitivity, and regional specialization, [Ca^2+^]o dynamics provide a versatile mechanism through which astrocytes and neurons coordinate activity. This perspective not only broadens our understanding of glial physiology but also opens new avenues for explaining emergent properties of neural circuits.

## 5. Ephaptic Coupling: A New Paradigm for Astrocyte–Neuron Communication

### 5.1. Theoretical Framework

Ephaptic coupling refers to a form of electrical communication in which activity in one cell influences neighboring cells through changes in the extracellular ionic milieu and electric fields, without the need for synaptic transmission or gap junctions. Unlike chemical synapses, where neurotransmitters bind to receptors, or gap junctions, which permit direct cytoplasmic continuity, ephaptic interactions operate through modifications in the shared extracellular environment [74,75,76,77,78]. This mechanism has long been considered in the context of neuronal communication, particularly in tightly packed axon bundles, but accumulating evidence now suggests that astrocytes may also contribute to such field-based signaling. The physical basis lies in ion concentration gradients and local field potentials generated in narrow extracellular spaces, where diffusion is restricted and local changes in charge density can influence membrane excitability of adjacent cells [77,79,80].

### 5.2. Astrocyte-Mediated Ephaptic Mechanisms

Astrocytes are ideally positioned to shape ephaptic communication through their capacity to regulate extracellular ion fluxes. Potassium buffering provides a classic example: by clearing excess K^+^ released during neuronal firing, astrocytes modulate the extracellular potential and thus influence neuronal excitability on rapid timescales [13,14,35,81,82]. Emerging data suggest that calcium may serve a similar role, as fluctuations in extracellular calcium concentration ([Ca^2+^]o) can directly affect neuronal channels and receptors. Astrocytes, by releasing or sequestering calcium through exchangers, ATP-driven pumps, and vesicular routes, are capable of driving these changes in restricted microdomains [9]. Other ions, including Na^+^ and Cl^−^, may also contribute, although their specific roles in astrocyte-mediated ephaptic signaling remain less well defined.

Geometry is central to the efficiency of this process. Ephaptic effects are strongest where the extracellular space is narrow, such as in synaptic clefts, perisynaptic astrocytic processes, and soma-to-soma contacts. In these compartments, the close apposition of astrocytic membranes to neurons amplifies the impact of local ionic shifts, while surface area considerations determine the extent of extracellular modulation. Recent studies on satellite astrocytes further emphasize that such structural arrangements are not random but are specialized to maximize the efficiency of field-based communication [44].

### 5.3. Functional Advantages

Ephaptic coupling offers several advantages compared with conventional communication modes. Because it relies on physical and ionic field effects, the signaling is extremely rapid, approaching the speed of electrical transmission without being constrained by receptor binding or vesicular release. This allows astrocytes to modulate neuronal excitability on timescales comparable to those of action potentials, bypassing the slower kinetics associated with gliotransmission [2,22,83,84,85,86]. Importantly, ephaptic mechanisms are inherently bidirectional: astrocytic activity can shape neuronal firing, while neuronal discharges can reciprocally alter astrocytic ionic states. This reciprocity enables a dynamic integration of network states, allowing local field changes to synchronize cellular ensembles across neuronal and glial populations. In doing so, ephaptic coupling extends the classical view of astrocytes as modulators of extracellular homeostasis toward a model in which they are active partners in fast information processing [22,76,87].

## 6. Pathological Implications and Disease Relevance

### 6.1. Disrupted Ca^2+^ Homeostasis in Neurological Disorders

Astrocytic calcium dynamics are highly sensitive to pathological stressors, and disturbances in these processes are now recognized as central features of several major neurological disorders [9,42].

In Alzheimer’s disease (AD), astrocytes display abnormal intracellular Ca^2+^ oscillations that amplify neuronal dysfunction. Amyloid-β aggregates alter the function of both metabotropic receptors and ion channels, producing exaggerated Ca^2+^ transients in astrocytes and interfering with extracellular calcium buffering [1,88,89]. These abnormalities destabilize gliotransmission, promote excitotoxic cascades, and impair amyloid clearance by disrupting Ca^2+^-dependent endocytosis. The net effect is a self-reinforcing cycle in which astrocytic Ca^2+^ dysregulation accelerates synaptic and cognitive decline [88,90,91,92,93,94,95,96,97].

In contrast to the widespread alterations seen in AD, Parkinson’s disease (PD) involves more selective vulnerability. Evidence points to striatal astrocytes, which normally regulate extracellular Ca^2+^ in close apposition to cholinergic interneurons, key modulators of striatal circuits [98,99,100,101,102]. Pathological α-synuclein accumulation disrupts astrocytic Ca^2+^ handling, weakening their ability to maintain ionic stability and altering the balance between dopaminergic and cholinergic signaling. This impairment may underlie both motor dysfunction and cognitive decline in PD [91,99,103,104,105,106,107].

Epilepsy provides perhaps the clearest example of extracellular calcium dysregulation. During seizures, [Ca^2+^]o drops rapidly as neurons fire synchronously and astrocytes attempt to buffer the surge in ion flux [6,35,104,108,109,110,111,112]. Impaired astrocytic Ca^2+^ handling compromises their ability to restore ionic homeostasis, prolonging hyperexcitability and facilitating recurrent ictal activity. Aberrant Ca^2+^ oscillations in astrocytes have also been implicated in lowering seizure thresholds, underscoring that astrocytic dysfunction is not merely reactive but actively drives network instability [35,111,113,114].

### 6.2. Developmental Disorders

Disrupted astrocytic Ca^2+^ dynamics also intersect with developmental conditions. In autism spectrum disorders (ASD), postmortem and animal studies suggest that astrocytes fail to fully mature, leading to abnormal Ca^2+^ signaling profiles. These deficits can impair synapse formation, pruning, and plasticity, processes in which finely tuned astrocytic calcium responses are essential. The consequence is altered circuit wiring that may underlie core behavioral phenotypes of ASD [113]. Similarly, intellectual disabilities have been linked to genetic mutations in Ca^2+^-handling proteins, including IP_3_ receptors, STIM/Orai components of store-operated entry, and various exchangers. In astrocytes, such mutations disrupt intracellular Ca^2+^ mobilization and extracellular modulation, impairing the support they normally provide to neuronal networks [4,16,35,115]. Because these pathways are crucial during early postnatal development, their disruption can result in long-lasting deficits in synaptic connectivity and cognitive function.

The main neurological and developmental disorders associated with astrocytic Ca^2+^ dysregulation, their cellular consequences, and translational implications are summarized in Table 3.

### 6.3. Therapeutic Implications

The recognition that astrocytic Ca^2+^ dynamics influence both intracellular and extracellular compartments opens new therapeutic perspectives. Pharmacological targeting of extracellular calcium handling is one promising direction. Modulators of calcium-sensing receptors (CaSR) could, in principle, recalibrate neuronal sensitivity to [Ca^2+^]o fluctuations, while interventions that stabilize astrocytic Ca^2+^ buffering might reduce excitability in epilepsy or normalize synaptic signaling in AD [16,42,116,117]. However, specificity remains a challenge: drugs that broadly alter extracellular calcium risk disrupting essential functions such as synaptic transmission and vascular tone. Another emerging avenue is astrocyte transplantation. Grafting healthy astrocytes into damaged circuits has shown promise in experimental models of epilepsy and neurodegeneration, restoring more physiological Ca^2+^ dynamics and rebalancing excitatory–inhibitory activity [23,24,35]. Future strategies may combine transplantation with genetic engineering to enhance Ca^2+^-handling capacity or tailor responses to disease contexts [118,119,120,121]. Together, these findings position astrocytic Ca^2+^ dysregulation as a convergent pathway across diverse diseases. Whether the disorder originates from protein aggregates, ion channel mutations, or developmental abnormalities, the end result often involves mismanaged Ca^2+^ signaling and disturbed astrocyte–neuron communication. Interventions that restore balanced Ca^2+^ flux hold the potential to stabilize networks and modify disease trajectories.

## 7. Technical Considerations and Methodological Advances

### 7.1. Current Techniques for Studying [Ca^2+^]o

The study of extracellular calcium dynamics has been propelled by major advances in experimental methodology. The earliest and most direct measurements relied on calcium-selective microelectrodes, which provided high temporal resolution but were limited in spatial precision. These electrodes can detect rapid [Ca^2+^]o fluctuations in the submillisecond range and remain valuable for quantifying fast changes during synaptic activity. However, their invasive nature, relatively large size compared to microdomains, and sensitivity to drift constrain their ability to capture localized events within restricted extracellular spaces [65,122,123].

Fluorescent indicators expanded the field by enabling visualization of [Ca^2+^]o changes with higher spatial resolution. Early small-molecule dyes were limited by diffusion and poor specificity for the extracellular compartment, but refinements in loading strategies and chemical modifications have improved their performance. Two-photon microscopy further increased spatial resolution and allowed investigators to map [Ca^2+^]o signals in intact tissue, including awake animals. Still, such indicators are typically less sensitive than electrodes for very rapid kinetics and may underestimate the amplitude of transient drops in calcium [124,125].

More recently, genetically encoded calcium indicators (GECIs) tailored for the extracellular space have emerged. By targeting GCaMP variants or engineered calcium-binding proteins to the plasma membrane or secreted domains, researchers can directly monitor extracellular calcium dynamics with cellular or even subcellular resolution [126,127,128]. These tools are still in development but offer the potential for long-term monitoring, cell-type specificity, and integration with optogenetics. Future iterations aim to combine high affinity with rapid kinetics, bridging the gap between electrode speed and imaging flexibility [9,125,129].

In parallel, fluorescence lifetime imaging microscopy (FLIM) has emerged as a complementary approach for studying calcium dynamics and cellular metabolism with nanosecond temporal precision. Unlike intensity-based imaging, FLIM measures the fluorescence decay time of Ca^2+^ indicators such as Fura-2 or GCaMP-based sensors, providing a quantitative, concentration-independent assessment of calcium fluctuations [130,131,132]. This method can distinguish free versus protein-bound Ca^2+^ pools and is less affected by dye loading or photobleaching artifacts. Importantly, FLIM enables the simultaneous assessment of calcium signaling and metabolic state through autofluorescence lifetime measurements of NADH and FAD, offering new insights into how astrocytic Ca^2+^ fluxes intersect with energy metabolism and redox balance. Although still technically demanding and limited in imaging depth, FLIM provides a powerful framework for linking ionic signaling with metabolic function in intact brain tissue [130,131,132].

### 7.2. Challenges and Limitations

Despite these advances, significant methodological challenges remain. Spatial resolution is a primary concern: extracellular calcium signals often occur in microdomains of a few tens of nanometers around synapses or perisynaptic astrocytic processes, far below the resolution of conventional microscopy [133,134]. As a result, bulk measurements risk averaging across compartments and obscuring localized dynamics. Diffusion artifacts further complicate interpretation, as extracellular calcium gradients are steep and can dissipate during experimental manipulation [9,135,136]. Temporal resolution is an equally pressing limitation. Millisecond-scale fluctuations may drive neuronal excitability, but most optical indicators cannot yet capture such rapid kinetics [137,138]. Distinguishing cause from effect also remains difficult, since changes in [Ca^2+^]o can arise from multiple sources, neuronal influx, astrocytic release, or buffering proteins, and current methods rarely allow for precise separation of these contributions. In addition, methodological constraints such as indicator buffering, phototoxicity, or unintended perturbation of the extracellular milieu can bias results. Thus, both spatial and temporal resolution remain limiting factors, constraining our ability to capture the full dynamics of [Ca^2+^]o [72,139,140].

The main experimental approaches to study extracellular calcium dynamics, their resolution limits, advantages, and typical applications are summarized in Table 4.

### 7.3. Future Technological Needs

Addressing these challenges will require the development of tools that combine high spatial and temporal resolution with minimal invasiveness. Chronic in vivo monitoring of [Ca^2+^]o in behaving animals is a key frontier, as it would allow researchers to link extracellular calcium dynamics directly to physiological states and behavioral outputs. Implantable biosensors or improved GECIs optimized for extracellular use may provide such capabilities [136,141,142,143,144,145]. Alongside experimental tools, computational approaches are expected to play a complementary role. Biophysical simulations can predict how synaptic activity, astrocytic buffering, and matrix composition interact to shape [Ca^2+^]o microdomains. Integrating these models with empirical measurements will help disentangle overlapping processes and identify causal mechanisms. Furthermore, machine learning approaches applied to large imaging datasets may uncover patterns of extracellular calcium dynamics that are difficult to detect by conventional analysis [146,147,148,149].

In sum, while significant progress has been made, the field still lacks a gold-standard technique capable of resolving extracellular calcium signals with the fidelity routinely achieved for intracellular Ca^2+^ imaging. Bridging this gap will be essential not only for a complete understanding of astrocyte–neuron communication but also for validating emerging concepts such as ephaptic coupling.

**Table 4:** Experimental tools to study extracellular Ca2+ dynamics.

Method	Spatial Resolution	Temporal Resolution	Advantages	Limitations	Typical Applications	Refs.
Calcium-selective microelectrodes	Limited (micrometer scale, relatively large tip size)	High (submillisecond range)	Direct quantitative measurements, high temporal resolution, minimal buffering effects, real-time monitoring	Invasive, sensitivity to drift and interference, poor spatial precision for microdomains, limited to point measurements	Detecting rapid [Ca^2+^]o fluctuations during synaptic activity and network events	[133,134,135,142]
Small-molecule fluorescent dyes	Moderate (micrometer scale)	Moderate (millisecond to second range, dependent on dye kinetics)	Improved spatial resolution over electrodes, visualization of spatial patterns, variety of affinity ranges available	Limited extracellular compartment specificity, potential dye diffusion and loading issues, phototoxicity during prolonged imaging, buffering effects at high concentrations; photobleaching	Mapping [Ca^2+^]o spatial distributions in tissue preparations and acute slices	[124,125,136,139]
Confocal microscopy	High (subcellular, ~200–500 nm lateral)	Moderate to high (milliseconds to seconds, depending on scanning mode)	Good optical sectioning; reduced out-of-focus fluorescence, compatible with multiple fluorophores	Limited penetration depth (<100 μm typically), phototoxicity and photobleaching; temporal resolution limited by scanning speed	Detailed spatial mapping of [Ca^2+^]o in superficial layers, co-localization studies	[49,124,139]
Two-photon microscopy	High (subcellular, ~300–700 nm lateral)	Variable (milliseconds to seconds, depending on scanning configuration and indicator)	Deep tissue penetration (up to ~1 mm), reduced phototoxicity and photobleaching, compatible with in vivo imaging in awake animals	May underestimate amplitude of rapid transient Ca^2+^ changes, slower scanning can miss fast events, expensive instrumentation, still subject to phototoxicity during chronic imaging	[Ca^2+^]o visualization in intact neural circuits, deep tissue and in vivo studies	[46,49,69,138,143]
GECIs for extracellular monitoring	Cellular to subcellular resolution	Developing (currently limited by indicator kinetics)	Long-term monitoring; cell-type and compartment specificity, genetic targeting, integration with optogenetics	Technology still under development, need to optimize affinity and kinetics for extracellular environment, potential buffering effects, phototoxicity during chronic imaging, expression level variability	Targeted monitoring of extracellular Ca^2+^ dynamics in specific cell populations or microdomains	[126,127,128,136,138]
Advanced scanning techniques (resonant scanners, acousto-optic deflectors, spinning disk)	High (subcellular)	Very high (submillisecond to millisecond range)	Enhanced temporal resolution, reduced motion artifacts, improved ability to capture fast Ca^2+^ transients	Hardware-dependent performance, may sacrifice signal-to-noise ratio for speed, specialized and expensive equipment	Capturing rapid [Ca^2+^]o dynamics during high-frequency neuronal activity	[143]
Implantable biosensors	Under development (potentially cellular scale)	Under development (potentially millisecond range)	Potential for chronic in vivo monitoring; minimal invasiveness; real-time physiological measurements	Emerging technology, biocompatibility concerns, calibration challenges, long-term stability issues	Linking [Ca^2+^]o dynamics to physiological states and behavior in freely moving animals	[126,128,136]
Computational modeling	Theoretical (nanometer to tissue scale, parameter-dependent)	Theoretical (microsecond to second range, timestep-dependent)	Predict microdomain dynamics inaccessible to current techniques, disentangle overlapping processes; identify causal mechanisms, test hypotheses in silico	Requires empirical validation, model assumptions and simplifications may limit accuracy, dependent on quality of input parameters	Biophysical simulations of synaptic activity, astrocytic buffering effects, and [Ca^2+^]o dynamics in restricted spaces	[8,41,74,135,141]

## 8. Future Directions and Unresolved Questions

Despite rapid progress, many aspects of astrocytic calcium signaling remain incompletely understood. At the molecular level, the identity of the precise transporters and exchangers that dominate extracellular calcium dynamics is still debated. While Na^+^–Ca^2+^ exchangers, Ca^2+^-ATPases, and vesicular pathways have been implicated [4,5,16], the relative contributions of each mechanism across different compartments of astrocytes and brain regions remain to be clarified. Another unresolved issue concerns the regulation of S100β and other calcium-binding proteins in the extracellular space [34,36]. Their release appears activity-dependent, yet the triggers and signaling consequences are poorly defined.

Establishing physiological relevance is equally important. Most studies so far have been carried out in cultured cells or acute slice preparations [6,150]. Verification in vivo, particularly under behaviorally relevant conditions, will be necessary to determine how extracellular calcium fluctuations shape neural circuit function. Linking these dynamics to cognition, sensory processing, or motor control remains a frontier challenge. From an evolutionary standpoint, it also remains unclear whether extracellular calcium signaling represents a conserved feature across species or whether it has emerged more recently in mammals alongside the expansion of astrocyte complexity. Developmental studies may help determine when these mechanisms first appear and how they contribute to circuit maturation.

Several emerging research areas hold promise for resolving these gaps. Multi-scale computational modeling can integrate molecular data with circuit-level dynamics, providing a framework to predict how calcium microdomains scale up to influence network behavior [151,152]. Optogenetic and chemogenetic tools now allow for cell-type–specific manipulations with high temporal precision, which can directly test causal relationships between astrocytic calcium fluxes and neuronal outcomes [16,148,153,154]. At the single-cell level, the growing appreciation of astrocyte heterogeneity suggests that individual subpopulations may contribute differently to calcium signaling. Combining single-cell transcriptomics with functional imaging may uncover distinct repertoires of transporters, receptors, and coupling strategies.

Translational opportunities are beginning to emerge. Extracellular calcium fluctuations, if reliably detected, could serve as biomarkers of brain health. Non-invasive monitoring strategies, ranging from improved calcium-sensitive dyes to biosensor-based imaging, may eventually allow clinicians to track these dynamics in patients. On the therapeutic side, selectively targeting astrocyte–neuron calcium interactions offers an avenue for precision medicine. Modulators of Ca^2+^ exchangers, blockers of aberrant gliotransmission, or interventions aimed at restoring buffering proteins like S100β could provide new treatment strategies for epilepsy, neurodegeneration, and neurodevelopmental disorders [6,33,53,112].

In summary, the field now stands at a pivotal stage. The essential molecular actors are only partially identified, the in vivo significance of rapid extracellular calcium changes is still emerging, and the translational potential has yet to be realized. Future work that bridges molecular, cellular, and systems-level approaches will be critical for turning these open questions into actionable insights.

## 9. Conclusions

Astrocytic calcium signaling represents a versatile system that operates across intracellular and extracellular compartments. Classical studies established the importance of intracellular Ca^2+^ oscillations, gliotransmitter release, and ion homeostasis. Building on this foundation, recent evidence reveals that extracellular calcium ([Ca^2+^]o) is not a static reservoir but a dynamic signaling mediator capable of influencing neuronal excitability within milliseconds. Mechanisms such as CaSR activation, ion channel modulation, and ephaptic coupling position astrocytes as active partners in both slow and rapid modes of neural communication.

This broadened perspective has major implications for physiology and disease. By linking intracellular dynamics to extracellular modulation, astrocytes contribute to network oscillations, synaptic plasticity, and circuit synchronization. Conversely, dysregulation of Ca^2+^ signaling is now recognized as a convergent pathway in Alzheimer’s disease, Parkinson’s disease, epilepsy, and developmental disorders, highlighting its relevance as a therapeutic target.

Although technical advances, ranging from Ca^2+^-selective electrodes to genetically encoded extracellular indicators, have opened new windows into these processes, significant challenges remain in resolving microdomain-scale and millisecond-range dynamics. Future progress will depend on combining improved tools with computational and translational approaches to clarify how extracellular calcium shapes neural circuits and how these pathways can be harnessed for clinical benefit.

In summary, astrocytes should be viewed not only as regulators of intracellular Ca^2+^ but also as architects of the extracellular ionic landscape. Recognizing this dual role reframes our understanding of neuron-glia interactions and provides new opportunities for therapeutic intervention.

## Figures and Tables

**Table 1:** Intracellular mechanisms of astrocytic Ca^2+^ signaling.

Mechanism/Source	Mode of Activation	Functional Output	References
IP_3_R (Inositol 1,4,5-trisphosphate receptors)	G-protein-coupled receptor activation → phospholipase C → IP_3_ production → ER Ca^2+^ release	Gliotransmitter release (glutamate, ATP, GABA, D-serine), metabolic coupling	[4,6,9,13,14]
SOCE (Store-operated calcium entry)	ER Ca^2+^ depletion → STIM sensor activation → Orai channel opening at ER-plasma membrane junctions	ER Ca^2+^ store replenishment, sustained Ca^2+^ signaling	[6,9,13]
mGluR (Metabotropic glutamate receptors)	Glutamate binding → phospholipase C activation → IP_3_-mediated Ca^2+^ mobilization	Gliotransmission, synaptic modulation	[4,8,9,12]
P2Y (Purinergic P2Y receptors)	ATP/ADP binding → phospholipase C activation → IP_3_-mediated Ca^2+^ mobilization	Gliotransmission, intercellular Ca^2+^ wave propagation	[4,9,13,14]
VGCC (Voltage-gated calcium channels)	Membrane depolarization → channel opening → Ca^2+^ influx	Localized Ca^2+^ entry, depolarization-linked responses	[5,9,13,14]
NCX (Na^+^-Ca^2+^ exchanger)	Reverse mode operation during elevated intracellular Na^+^ (e.g., after neurotransmitter uptake)	Ca^2+^ entry independent of ER stores, contribution to ionic homeostasis (with Na^+^,K^+^-ATPase)	[7,9,13]
Ionotropic receptors	Ligand binding (e.g., AMPA, NMDA, P2X) → direct Ca^2+^ influx	Microdomain signaling in fine processes	[4,9,13,14]

**Table 2:** Extracellular calcium signaling mechanisms and neuronal targets.

Neuronal Target	Mechanism of Sensitivity to [Ca^2+^]o	Functional Outcome	References
CaSR (Calcium-sensing receptor)	G-protein-coupled receptor activation by extracellular Ca^2+^ → intracellular cascades regulating ion channels, neurotransmitter release, and gene expression	Coupling of extracellular calcium availability to neuronal physiology	[39,57,60,61]
HCN channels (Hyperpolarization-activated cyclic nucleotide-gated)	Surface charge screening by Ca^2+^ ions → shielding of negative membrane charges → shifted voltage dependence	Altered pacemaker currents and excitability	[59,62]
SK/BK channels (Small/large-conductance Ca^2+^-activated K^+^ channels)	Indirect modulation through [Ca^2+^]o effects on local depolarization and intracellular Ca^2+^ entry	Modulation of afterhyperpolarization and firing patterns	[47,59,62]
NALCN (Sodium leak channel)	Proposed response to [Ca^2+^]o changes through mechanisms involving auxiliary subunits	Altered baseline excitability and membrane potential	[27,63,64]
Voltage-gated Na^+^/K^+^ channels	Screening of fixed negative charges by divalent Ca^2+^ alters gating of fast voltage-gated channels	Depolarized neuronal thresholds, increased excitability (with [Ca^2+^]o reduction)	[59,62,65]
NMDA receptors	Direct modulation by extracellular Ca^2+^ availability affecting receptor activity	Altered synaptic plasticity induction (LTP/LTD)	[30,47,66]
Presynaptic Ca^2+^ channels	[Ca^2+^]o changes alter driving force for Ca^2+^ entry during action potentials	Modified neurotransmitter release probability and short-term plasticity	[47,52,59]

**Table 3:** Pathological alterations in astrocytic Ca2+ signaling.

Disorder	Astrocytic Ca^2+^ Abnormality	Consequences for Neurons/Networks	Translational Implications	Refs.
Alzheimer’s disease (AD)	Abnormal intracellular Ca^2+^ oscillations; exaggerated Ca^2+^ transients; impaired extracellular Ca^2+^ buffering (due to amyloid-β effects on receptors/channels)	Destabilized gliotransmission; synaptic and cognitive decline	Excitotoxic cascades; impaired amyloid clearance via disrupted Ca^2+^-dependent endocytosis; self-reinforcing pathological cycle	[88,89,93,95,96]
Parkinson’s disease (PD)	Disrupted astrocytic Ca^2+^ handling in striatum (due to α-synuclein accumulation); weakened ability to maintain ionic stability particularly affecting astrocyte-cholinergic interneuron interactions	Altered balance between dopaminergic and cholinergic signaling; compromised striatal circuit function	Motor dysfunction and cognitive decline; selective vulnerability of striatal cholinergic interneurons	[99,100,101,103,104,105,106,107]
Epilepsy	Rapid [Ca^2+^]o drops during seizures; impaired astrocytic Ca^2+^ buffering; aberrant Ca^2+^ oscillations	Compromised ionic homeostasis restoration; prolonged hyperexcitability; facilitated recurrent ictal activity	Lowered seizure thresholds; network instability driven by astrocytic dysfunction; potential therapeutic target for stabilizing [Ca^2+^]o and restoring excitability balance	[35,82,110,111,114]
Autism spectrum disorders (ASD)	Immature astrocytic Ca^2+^ signaling profiles (due to failed astrocyte maturation)	Impaired synapse formation, pruning, and plasticity; altered circuit wiring	Synaptic maturation deficits leading to atypical circuit development and behavioral phenotypes	[36,67,68,83]
Intellectual disabilities (ID)	Mutations in Ca^2+^-handling proteins (IP_3_ receptors, STIM/Orai components, exchangers); disrupted intracellular Ca^2+^ mobilization and extracellular modulation	Impaired astrocytic support to neuronal networks; long-lasting deficits in synaptic connectivity	Cognitive dysfunction resulting in persistent impairments in learning and memory functions	[6,28,115]

## Data Availability

No new data were created or analyzed in this study. Data sharing is not applicable to this article.
